# 4-[(*E*)-(4-Eth­oxy­benzyl­idene)amino]­phenol

**DOI:** 10.1107/S1600536813012580

**Published:** 2013-05-18

**Authors:** Narissara Kaewmanee, Suchada Chantrapromma, Nawong Boonnak, Hoong-Kun Fun

**Affiliations:** a Department of Chemistry and Center of Excellence for Innovation in Chemistry, Faculty of Science, Prince of Songkla University, Hat-Yai, Songkhla 90112, Thailand; b Department of Chemistry, Faculty of Science, Prince of Songkla University, Hat-Yai, Songkhla 90112, Thailand; c Faculty of Traditional Thai Medicine, Prince of Songkla University, Hat-Yai, Songkhla 90112, Thailand; d X-ray Crystallography Unit, School of Physics, Universiti Sains Malaysia, 11800 USM, Penang, Malaysia; e Department of Pharmaceutical Chemistry, College of Pharmacy, King Saud University, PO Box 2457, Riyadh 11451, Saudi Arabia

## Abstract

The mol­ecule of the title compound, C_15_H_15_NO_2_, adopts a *trans* conformation with respect to the methyl­idene C=N bond and is twisted with a dihedral angle of 26.31 (5)° between the two substituted benzene rings. The eth­oxy group is almost coplanar with the bound benzene ring with a C—O—C—C torsion angle of −179.08 (9)°. In the crystal, mol­ecules are linked by O—H⋯N hydrogen bonds and weak C—H⋯O inter­actions into chains propagating in the [011] and [01-1] directions. C—H⋯π inter­actions are also present.

## Related literature

For standard bond lengths, see: Allen *et al.* (1987 ▶). For background to and applications of aza-stilbene, see: Cheng *et al.* (2010 ▶); da Silva *et al.* (2011 ▶); Kabir *et al.* (2008 ▶); Lu *et al.* (2012 ▶); Pavan *et al.* (2011 ▶). For related structures, see: Sun *et al.* (2011 ▶); Wang (2009 ▶).
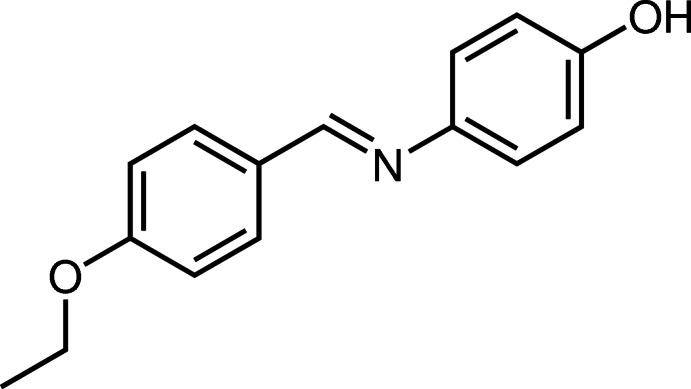


## Experimental

### 

#### Crystal data

C_15_H_15_NO_2_
*M* *_r_* = 241.28
Orthorhombic, 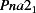

*a* = 18.6882 (11) Å
*b* = 10.7420 (6) Å
*c* = 6.3186 (4) Å
*V* = 1268.45 (13) Å^3^
*Z* = 4
Mo *K*α radiation
μ = 0.08 mm^−1^
*T* = 100 K
0.59 × 0.27 × 0.25 mm

#### Data collection

Bruker SMART APEXII CCD area-detector diffractometer
Absorption correction: multi-scan (*SADABS*; Bruker, 2009 ▶) *T*_min_ = 0.952, *T*_max_ = 0.979
16922 measured reflections
2382 independent reflections
2319 reflections with *I* > 2σ(*I*)
*R*_int_ = 0.025

#### Refinement

*R*[*F* ^2^ > 2σ(*F* ^2^)] = 0.030
*wR*(*F* ^2^) = 0.086
*S* = 1.10
2382 reflections
168 parameters
1 restraint
H atoms treated by a mixture of independent and constrained refinement
Δρ_max_ = 0.35 e Å^−3^
Δρ_min_ = −0.19 e Å^−3^

### 

Data collection: *APEX2* (Bruker, 2009 ▶); cell refinement: *SAINT* (Bruker, 2009 ▶); data reduction: *SAINT*; program(s) used to solve structure: *SHELXTL* (Sheldrick, 2008 ▶); program(s) used to refine structure: *SHELXTL*; molecular graphics: *SHELXTL*; software used to prepare material for publication: *SHELXTL* and *PLATON* (Spek, 2009 ▶).

## Supplementary Material

Crystal structure: contains datablock(s) global, I. DOI: 10.1107/S1600536813012580/rz5061sup1.cif

Structure factors: contains datablock(s) I. DOI: 10.1107/S1600536813012580/rz5061Isup2.hkl

Supplementary material file. DOI: 10.1107/S1600536813012580/rz5061Isup3.cml

Additional supplementary materials:  crystallographic information; 3D view; checkCIF report

## Figures and Tables

**Table 1:** Hydrogen-bond geometry (Å, °) *Cg*1 is the centroid of the C8–C13 ring.

*D*—H⋯*A*	*D*—H	H⋯*A*	*D*⋯*A*	*D*—H⋯*A*
O2—H1*O*2⋯N1^i^	0.83 (2)	1.87 (2)	2.6971 (12)	172 (2)
C6—H6*A*⋯O2^ii^	0.95	2.51	3.3956 (14)	156
C9—H9*A*⋯O2^iii^	0.95	2.38	3.3229 (13)	171
C14—H14*B*⋯*Cg*1^iv^	0.99	2.90	3.7668 (12)	147

Symmetry codes: (i) 
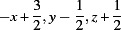
; (ii) 
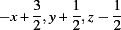
; (iii) 
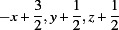
; (iv) 
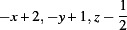
.

**Fig. 1.**
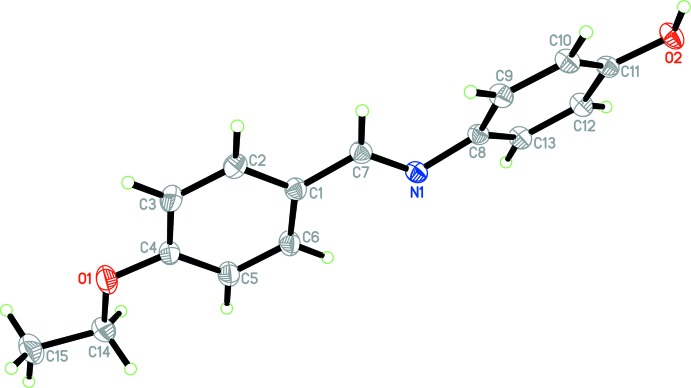
The asymmetric unit of the title compound showing 65% probability displacement ellipsoids.

**Fig. 2.**
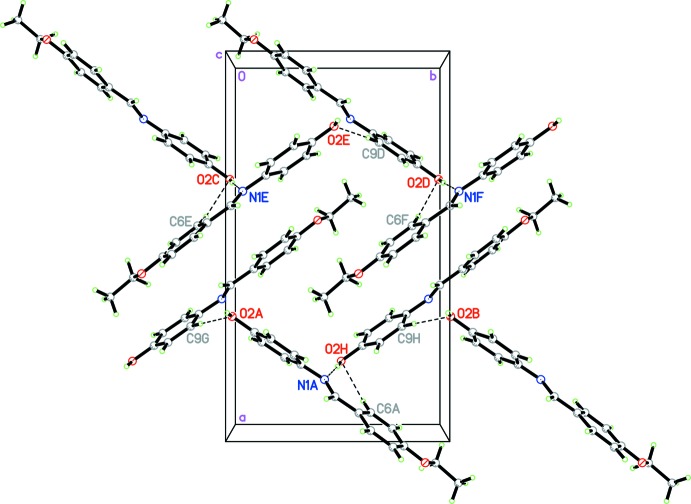
Crystal packing of the title compound viewed along the c axis. Hydrogen bonds are drawn as dashed lines.

## supplementary crystallographic information

### Comment

Aza-stilbene derivatives derived from the reaction of an aldehyde with hydrazine have been shown to possess potent biological activities such as antibacterial (Kabir *et al.*, 2008), antifungal (da Silva *et al.*, 2011), antimycobacterium tuberculosis (Pavan *et al.*, 2011) and antioxidation (Cheng *et al.*, 2010; Lu *et al.*, 2012) properties. These interesting biological activities of aza-stilbene led us to synthesize the title compound, (I), and study its antibacterial activity. Our antibacterial assay showed that (I) exhibits moderate activity against *Salmonella typhi* with the minimun inhibition concentration (MIC) value of 18.75 µg/ml. We report here the crystal structure of the title compound.

The molecule of (I) (Fig. 1), C_15_H_15_NO_2_, is twisted and exists in a *trans* configuration with respect to the methylidene C7═N1 double bond [1.2867 (13) Å] with the torsion angle C8–N1–C7–C1 = 179.23 (8)°. The dihedral angle between the two substituted benzene rings is 26.31 (5)°. The ethoxy group is co-planar with the bound benzene ring with the *r.m.s*. deviation of 0.0155 (1) Å for the nine non H-atoms and the C4–O1–C14–C15 angle is -179.08 (9)°. The bond distances are within the normal range (Allen *et al.*, 1987) and are in agreement with those reported for related structures (Sun *et al.*, 2011; Wang, 2009).

In the crystal structure (Fig. 2), the molecules are linked by O–H···N hydrogen bond and weak C—H···O interactions (Table 1) into chains propagating in the [011] and [011] directions. C—H···π interactions between the ethoxy group and the hydroxy substituted rings are also present (Table 1).

### Experimental

The title compound (I) was prepared by dissolving 4-benzylideneaniline (5 mmol, 0.50 g) in ethanol (30 ml) and 4-ethoxybenzaldehyde (5 mmol, 0.70 ml) was slowly added with stirring. The solution was stirred at room temperature for around 3 hr yielding a white solid, which was filtered off and washed with cold ethanol and dried in air. Colourless block-shaped single crystals of (I) suitable for X-ray structure determination were recrystallized from methanol by slow evaporation at room temperature after several days, M. p. 470–472 K.

### Refinement

The hydroxyl H atom was located in a difference map and refined isotropically. The remaining H atoms were fixed geometrically and allowed to ride on their parent atoms, with d(C—H) = 0.95 Å for aromatic and CH, 0.99 Å for CH_2_ and 0.98 Å for CH_3_ atoms. The *U*_iso_ values were constrained to be 1.5*U*_eq_ of the carrier atom for methyl H atoms and 1.2*U*_eq_ for the remaining H atoms. A rotating group model was used for the methyl group. 1762 Friedel pairs were merged as there is insufficient anomalous dispersion to determine the absolute structure since Mo radiation was used for data collection.

### Crystal data

**Table:**

C_15_H_15_NO_2_	*D*_x_ = 1.263 Mg m^−^^3^
*M**_r_* = 241.28	Melting point = 470–472 K
Orthorhombic, *P**n**a*2_1_	Mo *K*α radiation, λ = 0.71073 Å
Hall symbol: P 2c -2n	Cell parameters from 2382 reflections
*a* = 18.6882 (11) Å	θ = 2.2–32.0°
*b* = 10.7420 (6) Å	µ = 0.08 mm^−^^1^
*c* = 6.3186 (4) Å	*T* = 100 K
*V* = 1268.45 (13) Å^3^	Block, colourless
*Z* = 4	0.59 × 0.27 × 0.25 mm
*F*(000) = 512	

### Data collection

**Table:**

Bruker SMART APEXII CCD area-detector diffractometer	2382 independent reflections
Radiation source: fine-focus sealed tube	2319 reflections with *I* > 2σ(*I*)
Graphite monochromator	*R*_int_ = 0.025
Detector resolution: 8.33 pixels mm^-1^	θ_max_ = 32.0°, θ_min_ = 2.2°
ω scans	*h* = −27→27
Absorption correction: multi-scan (*SADABS*; Bruker, 2009)	*k* = −16→15
*T*_min_ = 0.952, *T*_max_ = 0.979	*l* = −9→9
16922 measured reflections	

### Refinement

**Table:**

Refinement on *F*^2^	Primary atom site location: structure-invariant direct methods
Least-squares matrix: full	Secondary atom site location: difference Fourier map
*R*[*F*^2^ > 2σ(*F*^2^)] = 0.030	Hydrogen site location: inferred from neighbouring sites
*wR*(*F*^2^) = 0.086	H atoms treated by a mixture of independent and constrained refinement
*S* = 1.10	*w* = 1/[σ^2^(*F*_o_^2^) + (0.0562*P*)^2^ + 0.1397*P*] where *P* = (*F*_o_^2^ + 2*F*_c_^2^)/3
2382 reflections	(Δ/σ)_max_ = 0.001
168 parameters	Δρ_max_ = 0.35 e Å^−^^3^
1 restraint	Δρ_min_ = −0.19 e Å^−^^3^

### Special details

**Table:**

Experimental. The data was collected with the Oxford Cryosystem Cobra low-temperature attachment.
Geometry. All e.s.d.'s (except the e.s.d. in the dihedral angle between two l.s. planes) are estimated using the full covariance matrix. The cell e.s.d.'s are taken into account individually in the estimation of e.s.d.'s in distances, angles and torsion angles; correlations between e.s.d.'s in cell parameters are only used when they are defined by crystal symmetry. An approximate (isotropic) treatment of cell e.s.d.'s is used for estimating e.s.d.'s involving l.s. planes.
Refinement. Refinement of *F*^2^ against ALL reflections. The weighted *R*-factor *wR* and goodness of fit *S* are based on *F*^2^, conventional *R*-factors *R* are based on *F*, with *F* set to zero for negative *F*^2^. The threshold expression of *F*^2^ > σ(*F*^2^) is used only for calculating *R*-factors(gt) *etc*. and is not relevant to the choice of reflections for refinement. *R*-factors based on *F*^2^ are statistically about twice as large as those based on *F*, and *R*- factors based on ALL data will be even larger.

### Fractional atomic coordinates and isotropic or equivalent isotropic displacement parameters (Å^2^)

**Table:**

	*x*	*y*	*z*	*U*_iso_*/*U*_eq_	
O1	1.07262 (4)	0.90374 (7)	0.65280 (16)	0.02092 (17)	
O2	0.68429 (4)	0.01683 (7)	0.72655 (13)	0.01662 (15)	
H1O2	0.6732 (11)	0.000 (2)	0.851 (4)	0.038 (5)*	
N1	0.85372 (4)	0.43914 (7)	0.61658 (15)	0.01367 (16)	
C1	0.93803 (5)	0.59761 (9)	0.72367 (18)	0.01420 (18)	
C2	0.97668 (5)	0.64779 (9)	0.89339 (19)	0.01686 (19)	
H2A	0.9725	0.6113	1.0298	0.020*	
C3	1.02104 (6)	0.75025 (10)	0.86479 (19)	0.0181 (2)	
H3A	1.0468	0.7835	0.9813	0.022*	
C4	1.02770 (5)	0.80430 (9)	0.66463 (19)	0.01595 (19)	
C5	0.98933 (6)	0.75587 (10)	0.49369 (19)	0.01789 (19)	
H5A	0.9934	0.7928	0.3576	0.021*	
C6	0.94517 (6)	0.65320 (10)	0.52393 (19)	0.01713 (19)	
H6A	0.9194	0.6201	0.4072	0.021*	
C7	0.89365 (5)	0.48741 (8)	0.76077 (17)	0.01413 (18)	
H7A	0.8944	0.4500	0.8970	0.017*	
C8	0.81221 (5)	0.33104 (9)	0.65972 (16)	0.01283 (17)	
C9	0.78245 (5)	0.30394 (9)	0.85792 (16)	0.01439 (18)	
H9A	0.7915	0.3572	0.9750	0.017*	
C10	0.73949 (5)	0.19880 (9)	0.88339 (16)	0.01468 (17)	
H10A	0.7190	0.1811	1.0176	0.018*	
C11	0.72652 (5)	0.11939 (8)	0.71249 (17)	0.01325 (17)	
C12	0.75683 (6)	0.14583 (9)	0.51485 (17)	0.01527 (18)	
H12A	0.7489	0.0913	0.3988	0.018*	
C13	0.79848 (5)	0.25179 (9)	0.48866 (16)	0.01442 (17)	
H13A	0.8178	0.2707	0.3534	0.017*	
C14	1.08402 (6)	0.95958 (10)	0.4482 (2)	0.0215 (2)	
H14A	1.0383	0.9914	0.3904	0.026*	
H14B	1.1037	0.8975	0.3482	0.026*	
C15	1.13632 (6)	1.06515 (11)	0.4787 (3)	0.0285 (3)	
H15A	1.1448	1.1065	0.3427	0.043*	
H15B	1.1816	1.0323	0.5335	0.043*	
H15C	1.1165	1.1253	0.5795	0.043*	

### Atomic displacement parameters (Å^2^)

**Table:**

	*U*^11^	*U*^22^	*U*^33^	*U*^12^	*U*^13^	*U*^23^
O1	0.0213 (3)	0.0180 (3)	0.0234 (4)	−0.0072 (3)	−0.0018 (3)	−0.0007 (3)
O2	0.0230 (3)	0.0138 (3)	0.0131 (3)	−0.0056 (2)	0.0018 (3)	−0.0011 (3)
N1	0.0156 (3)	0.0116 (3)	0.0138 (4)	−0.0003 (3)	0.0002 (3)	0.0000 (3)
C1	0.0144 (4)	0.0133 (3)	0.0148 (4)	−0.0003 (3)	−0.0007 (3)	−0.0011 (3)
C2	0.0177 (4)	0.0183 (4)	0.0146 (4)	−0.0008 (3)	−0.0029 (4)	−0.0003 (4)
C3	0.0174 (4)	0.0193 (4)	0.0176 (5)	−0.0023 (3)	−0.0036 (4)	−0.0023 (4)
C4	0.0146 (4)	0.0140 (4)	0.0193 (5)	−0.0013 (3)	−0.0018 (4)	−0.0007 (4)
C5	0.0199 (4)	0.0175 (4)	0.0162 (5)	−0.0045 (3)	−0.0025 (4)	0.0009 (4)
C6	0.0188 (4)	0.0176 (4)	0.0150 (4)	−0.0044 (3)	−0.0017 (4)	−0.0002 (4)
C7	0.0153 (4)	0.0132 (4)	0.0139 (4)	−0.0001 (3)	0.0000 (3)	−0.0002 (3)
C8	0.0144 (3)	0.0114 (3)	0.0126 (4)	0.0002 (3)	−0.0005 (3)	0.0001 (3)
C9	0.0184 (4)	0.0128 (4)	0.0119 (4)	−0.0005 (3)	0.0006 (3)	−0.0015 (3)
C10	0.0192 (4)	0.0137 (4)	0.0111 (4)	−0.0010 (3)	0.0012 (4)	−0.0007 (3)
C11	0.0161 (4)	0.0113 (3)	0.0123 (4)	−0.0001 (3)	0.0005 (3)	−0.0001 (3)
C12	0.0196 (4)	0.0143 (4)	0.0119 (4)	−0.0018 (3)	0.0011 (4)	−0.0015 (3)
C13	0.0181 (4)	0.0146 (4)	0.0106 (4)	−0.0011 (3)	0.0009 (4)	−0.0010 (3)
C14	0.0199 (4)	0.0176 (4)	0.0269 (6)	−0.0038 (3)	−0.0014 (4)	0.0044 (4)
C15	0.0236 (5)	0.0201 (5)	0.0420 (8)	−0.0071 (4)	0.0000 (5)	0.0019 (5)

### Geometric parameters (Å, º)

**Table:**

O1—C4	1.3605 (11)	C7—H7A	0.9500
O1—C14	1.4412 (16)	C8—C13	1.3996 (14)
O2—C11	1.3581 (11)	C8—C9	1.4009 (14)
O2—H1O2	0.83 (3)	C9—C10	1.3951 (13)
N1—C7	1.2867 (13)	C9—H9A	0.9500
N1—C8	1.4228 (12)	C10—C11	1.3973 (14)
C1—C2	1.4008 (15)	C10—H10A	0.9500
C1—C6	1.4026 (16)	C11—C12	1.4004 (15)
C1—C7	1.4643 (13)	C12—C13	1.3887 (13)
C2—C3	1.3898 (14)	C12—H12A	0.9500
C2—H2A	0.9500	C13—H13A	0.9500
C3—C4	1.3972 (16)	C14—C15	1.5094 (15)
C3—H3A	0.9500	C14—H14A	0.9900
C4—C5	1.3970 (15)	C14—H14B	0.9900
C5—C6	1.3906 (14)	C15—H15A	0.9800
C5—H5A	0.9500	C15—H15B	0.9800
C6—H6A	0.9500	C15—H15C	0.9800
			
C4—O1—C14	117.86 (9)	C10—C9—C8	119.99 (9)
C11—O2—H1O2	112.7 (15)	C10—C9—H9A	120.0
C7—N1—C8	120.62 (9)	C8—C9—H9A	120.0
C2—C1—C6	118.43 (9)	C9—C10—C11	120.33 (9)
C2—C1—C7	118.72 (10)	C9—C10—H10A	119.8
C6—C1—C7	122.83 (9)	C11—C10—H10A	119.8
C3—C2—C1	120.85 (11)	O2—C11—C10	123.06 (9)
C3—C2—H2A	119.6	O2—C11—C12	117.25 (9)
C1—C2—H2A	119.6	C10—C11—C12	119.68 (9)
C2—C3—C4	120.00 (10)	C13—C12—C11	119.95 (9)
C2—C3—H3A	120.0	C13—C12—H12A	120.0
C4—C3—H3A	120.0	C11—C12—H12A	120.0
O1—C4—C5	124.52 (10)	C12—C13—C8	120.62 (9)
O1—C4—C3	115.52 (9)	C12—C13—H13A	119.7
C5—C4—C3	119.96 (9)	C8—C13—H13A	119.7
C6—C5—C4	119.59 (10)	O1—C14—C15	107.10 (11)
C6—C5—H5A	120.2	O1—C14—H14A	110.3
C4—C5—H5A	120.2	C15—C14—H14A	110.3
C5—C6—C1	121.18 (10)	O1—C14—H14B	110.3
C5—C6—H6A	119.4	C15—C14—H14B	110.3
C1—C6—H6A	119.4	H14A—C14—H14B	108.5
N1—C7—C1	122.74 (9)	C14—C15—H15A	109.5
N1—C7—H7A	118.6	C14—C15—H15B	109.5
C1—C7—H7A	118.6	H15A—C15—H15B	109.5
C13—C8—C9	119.41 (9)	C14—C15—H15C	109.5
C13—C8—N1	116.65 (9)	H15A—C15—H15C	109.5
C9—C8—N1	123.87 (9)	H15B—C15—H15C	109.5
			
C6—C1—C2—C3	0.01 (15)	C6—C1—C7—N1	−5.42 (15)
C7—C1—C2—C3	178.24 (9)	C7—N1—C8—C13	−150.56 (9)
C1—C2—C3—C4	−0.20 (15)	C7—N1—C8—C9	32.46 (13)
C14—O1—C4—C5	−2.99 (14)	C13—C8—C9—C10	−0.07 (14)
C14—O1—C4—C3	177.23 (9)	N1—C8—C9—C10	176.84 (9)
C2—C3—C4—O1	−179.72 (9)	C8—C9—C10—C11	0.67 (14)
C2—C3—C4—C5	0.48 (15)	C9—C10—C11—O2	−178.92 (9)
O1—C4—C5—C6	179.64 (9)	C9—C10—C11—C12	−0.06 (15)
C3—C4—C5—C6	−0.58 (16)	O2—C11—C12—C13	177.76 (9)
C4—C5—C6—C1	0.40 (16)	C10—C11—C12—C13	−1.16 (15)
C2—C1—C6—C5	−0.12 (15)	C11—C12—C13—C8	1.78 (15)
C7—C1—C6—C5	−178.27 (9)	C9—C8—C13—C12	−1.16 (14)
C8—N1—C7—C1	179.23 (8)	N1—C8—C13—C12	−178.29 (9)
C2—C1—C7—N1	176.43 (9)	C4—O1—C14—C15	−179.08 (9)

### Hydrogen-bond geometry (Å, º)

*Cg*1 is the centroid of the C8–C13 ring.

**Table:**

*D*—H···*A*	*D*—H	H···*A*	*D*···*A*	*D*—H···*A*
O2—H1*O*2···N1^i^	0.83 (2)	1.87 (2)	2.6971 (12)	172 (2)
C6—H6*A*···O2^ii^	0.95	2.51	3.3956 (14)	156
C9—H9*A*···O2^iii^	0.95	2.38	3.3229 (13)	171
C14—H14*B*···*Cg*1^iv^	0.99	2.90	3.7668 (12)	147

Symmetry codes: (i) −*x*+3/2, *y*−1/2, *z*+1/2; (ii) −*x*+3/2, *y*+1/2, *z*−1/2; (iii) −*x*+3/2, *y*+1/2, *z*+1/2; (iv) −*x*+2, −*y*+1, *z*−1/2.

## References

[bb1] Allen, F. H., Kennard, O., Watson, D. G., Brammer, L., Orpen, A. G. & Taylor, R. (1987). *J. Chem. Soc. Perkin Trans. 2*, pp. S1–S19.

[bb2] Bruker (2009). *APEX2*, *SAINT* and *SADABS*, Bruker AXS Inc., Madison, Wisconsin, USA.

[bb3] Cheng, L.-X., Tang, J.-J., Luo, H., Jin, X.-L., Dai, F., Yang, J., Qian, Y.-P., Li, X.-Z. & Zhou, B. (2010). *Bioorg. Med. Chem. Lett.* **20**, 2417–2420. 10.1016/j.bmcl.2010.03.039 20346660

[bb4] Kabir, M. S., Engelbrecht, K., Polanowski, R., Krueger, S. M., Ignasiak, R., Rott, M., Schwan, W. R., Stemper, M. E., Reed, K. D., Sherman, D., Cook, J. M. & Monte, A. (2008). *Bioorg. Med. Chem. Lett.* **18**, 5745–5749. 10.1016/j.bmcl.2008.09.085 PMC7127099 18849164

[bb5] Lu, J., Li, C., Chai, Y.-F., Yang, D.-Y. & Sun, C.-R. (2012). *Bioorg. Med. Chem. Lett.* **22**, 5744–5747. 10.1016/j.bmcl.2012.06.026 22832313

[bb6] Pavan, F. R., de Carvalho, G. S. G., da Silva, A. D. & Leite, C. Q. F. (2011). *Sci. World J.* **11**, 1113–1119. 10.1100/tsw.2011.110 PMC5720119 21623457

[bb7] Sheldrick, G. M. (2008). *Acta Cryst.* A**64**, 112–122. 10.1107/S0108767307043930 18156677

[bb8] Silva, C. M. da, da Silva, D. L., Martins, C. V. B., de Resende, M. A., Dias, E. S., Magalhaes, T. F. F., Rodrigues, L. P., Sabino, A. A., Alves, R. B. & de Fatima, A. (2011). *Chem. Biol. Drug Des.* **78**, 810–815. 10.1111/j.1747-0285.2011.01185.x 21756287

[bb9] Spek, A. L. (2009). *Acta Cryst.* D**65**, 148–155. 10.1107/S090744490804362X PMC2631630 19171970

[bb10] Sun, L.-X., Yu, Y.-D. & Wei, G.-Y. (2011). *Acta Cryst.* E**67**, o1578. 10.1107/S1600536811019921 PMC3152012 21836989

[bb11] Wang, C.-Y. (2009). *Acta Cryst.* E**65**, o741. 10.1107/S1600536809008046 PMC2969002 21582474

